# Short-term causal effects of common treatments in ambulatory children and young adults with cerebral palsy: three machine learning estimates

**DOI:** 10.1038/s41598-022-11875-5

**Published:** 2022-05-12

**Authors:** Michael H. Schwartz, Andrew J. Ries, Andrew G. Georgiadis

**Affiliations:** 1 Gillette Children’s Specialty Healthcare, St. Paul, USA; 2 Department of Orthopedic Surgery, University of Minnesota, Minneapolis, USA; 3 Center for Gait and Motion Analysis, 205 University Ave. SE, St. Paul, MN 55101 USA

**Keywords:** Outcomes research, Paediatric research, Diseases of the nervous system, Computational biology and bioinformatics

## Abstract

Orthopedic and neurological impairments (e.g., muscle contractures, spasticity) are often treated in children and young adults with cerebral palsy (CP). Due to challenges arising from combinatorics, research funding priorities, and medical practicalities, and despite extensive study, the evidence base is weak. Our goal was to estimate the short-term effectiveness of 13 common orthopedic and neurological treatments at four different levels of outcome in children and young adults diagnosed with CP. The outcome levels considered were body structures, specific gait kinematic deviations, overall gait kinematic deviations, and functional mobility. We used three well-establish causal inference approaches (direct matching, virtual twins, and Bayesian causal forests) and a large clinical gait analysis database to estimate the average treatment effect on the treated (ATT). We then examined the effectiveness across treatments, methods, and outcome levels. The dataset consisted of 2851 limbs from 933 individuals (some individuals underwent multiple treatment episodes). Current treatments have medium effects on body structures, but modest to minimal effects on gait and functional mobility. The median ATT of 13 common treatments in children and young adults with CP, measured as Cohen’s D, bordered on medium at the body structures level (median [IQR] = 0.42 [0.05, 0.60]) and became smaller as we moved along the causal chain through specific kinematic deviations (0.21 [0.01, 0.33]), overall kinematic deviations (0.09 [0.03, 0.19]), and functional mobility (-0.01 [-0.06, 0.13]). Further work is needed to understand the source of heterogeneous treatment effects, which are large in this patient population. Replication or refutation of these findings by other centers will be valuable to establish the generalizability of these results and for benchmarking of best practices.

## Introduction

### Cerebral palsy and orthopedic deformity

There are approximately 750,000 people in the United States currently diagnosed with cerebral palsy (CP), and 10,000 newly diagnosed individuals each year^1–3^. Medical costs for children diagnosed with CP are 10–26 times higher than for typically developing children^4^. Around 70% of individuals diagnosed with CP are ambulatory^5^. The primary neurological impairments commonly found in individuals with CP include spasticity, reduced motor control, and weakness. Over time, these neurological impairments often lead to orthopedic deformity.

Neurological manifestations of CP and subsequent orthopedic deformities are frequently treated by surgery or neurotoxin injections. The rationale for treatment is that impairment at the body structure level impacts gait and mobility, which interferes with activities and participation, thereby contributing to reduced quality of life. Thus, by intervening at the body structures level, it is hoped that changes will propagate through the causal chain, ultimately leading to improvements in activities and participation and quality of life. The patients seen for gait analysis at our center are ambulatory, and skew towards bilateral involvement. As a result, treatment goals are usually multi-level, including (1) body structure goals, such as reducing excessive femoral anteversion or spasticity, (2) specific gait kinematic goals, such as correcting in-toeing, (3) overall gait kinematic goals, such as improving the overall walking motion, and (4) functional mobility goals, such as improving activity and participation related tasks like stair climbing.

Because problems do not occur in isolation, surgery in children and young adults with CP is often executed at multiple levels during a single operation (single-event multi-level surgery—SEMLS). This makes it hard to estimate the isolated impact of an individual surgery. For one thing, the 13 relatively common surgeries considered in this study can be combined in 8192 unique ways. Furthermore, there is limited funding available to study the effectiveness of established treatments in CP^6^. Finally, given how well-established most of the treatments in CP are, it would be difficult to find patients and surgeons willing to participate in randomized controlled trials (RCTs). There have been successful RCTs conducted in CP. For example, examining rectus femoris transfer, selective dorsal rhizotomy, and individualized care plans based on gait analysis^7–9^. However, much of what we know about treatment outcome in CP is based on observational studies. The design of these studies is often insufficient to establish strong evidence. Examples include not comparing to a control group, relying on case studies, and deferring to expert opinion^10^.

### Causal inference

Observational studies are susceptible to selection bias. Patients receiving different treatments are not randomized, and thus differ in their baseline characteristics. In addition, patients are generally chosen for treatments based on a doctor’s reasonable belief that the patient will either benefit from the treatment, fare poorly without a treatment, or both. This last element, known as targeted selection, causes important but often unrecognized problems when estimating treatment outcomes in observational studies^11,12^. Despite these challenges, it is critical that we understand the effectiveness of treatments for individuals with CP.

The RCT design is the gold standard for establishing causal inference, but it is not the only option^13^. There are many statistical and machine learning methods that can be used to estimate treatment effects^14^. These methods rely on adjusting for and regressing on important covariates that determine both treatment assignment and treatment outcome. Causal inference has gained popularity over the years and has been validated by reproducing the results of RCTs and deriving accurate effects from synthetic data. For example, in the context of CP treatment, we have recently shown that a standard causal inference technique can accurately and precisely estimate the effects of rectus femoris transfer compared to an RCT^15^. We have used similar methods to estimate the effect of SEMLS^16^.

In the present study we will use three modern causal inference methods: direct matching (DM), virtual twins (VT), and Bayesian Causal Forests (BCF) to estimate average treatment effects on the treated (ATT) for 13 common treatments in children and young adults with CP. We briefly describe these methods below. We will estimate outcomes at four levels: body structures, specific gait kinematic deviations, overall gait kinematic deviations, and functional mobility. The DM model will generate a matched subset of treated observations. We will then use this matched subset to estimate an ATT with both the VT and BCF models. We will also estimate an ATT from the VT and BCF models using the entire set of treated observations. By comparing the ATT estimates obtained from the matched subset to those obtained using all treated observations, we will identify possible bias due to omitted observations. We will consider the three models’ estimates together when interpreting the results. In doing so we will obtain a robust picture of the overall effectiveness of common treatments used for correcting deformity in children and young adults with CP.

## Results

Relevant summary data are provided in this section. In support of transparency and thoroughness, a detailed report for each of the 13 treatments is available as electronic addendum to this manuscript.

### Clinical profile

The data for this analysis were from limbs of patients seen for clinical evaluation in our gait analysis laboratory between 2003 and 2020 (inclusive). These patients were ambulatory, and evaluated prior to treatment. As a result, they do not reflect the entire population of individuals with CP. The dataset consisted of 2851 limbs from 933 individuals (some individuals underwent multiple treatment episodes). After excluding observations with missing covariates or Functional Assessment Questionnaire Transform (FAQt) values we were left with 2502 limbs from 837 individuals (Table 1). There was no noticeable pattern to the missing data. The main culprits were missing survey data (FAQt, N_miss_ = 224) due to typical non-response rates, and maximum passive ankle dorsiflexion with the knee extended (ANK_DORS_0, N_miss_ = 144) which cannot be measured in the presence of severe knee flexion contractures. No other variable was missing for more than 24 limbs. Note that the final number of observations for each treatment will be smaller than 2502, and will vary slightly between treatments, due to missing treatment-specific outcome data.

**Table 1:** Observation (limb) characteristics.

Covariate	Value
Age in years (mean (SD))	9.4 (3.7)
Sex (N male (%))	1453 (58.1)
Follow up years (mean (SD))	1.5 (0.4)
**Topographic classification (N (%))**	
Hemiplegia	202 (8.1)
Diplegia	1634 (65.3)
Triplegia	416 (16.6)
Quadriplegia	250 (10.0)
**GMFCS level (N (%))**	
I	584 (23.3)
II	844 (33.7)
III	552 (22.1)
IV	19 (0.8)
Missing	503 (20.1)

Limb is the fundamental unit of observation, so values are reported per limb.

### Treatment effects

**Figure 1:**
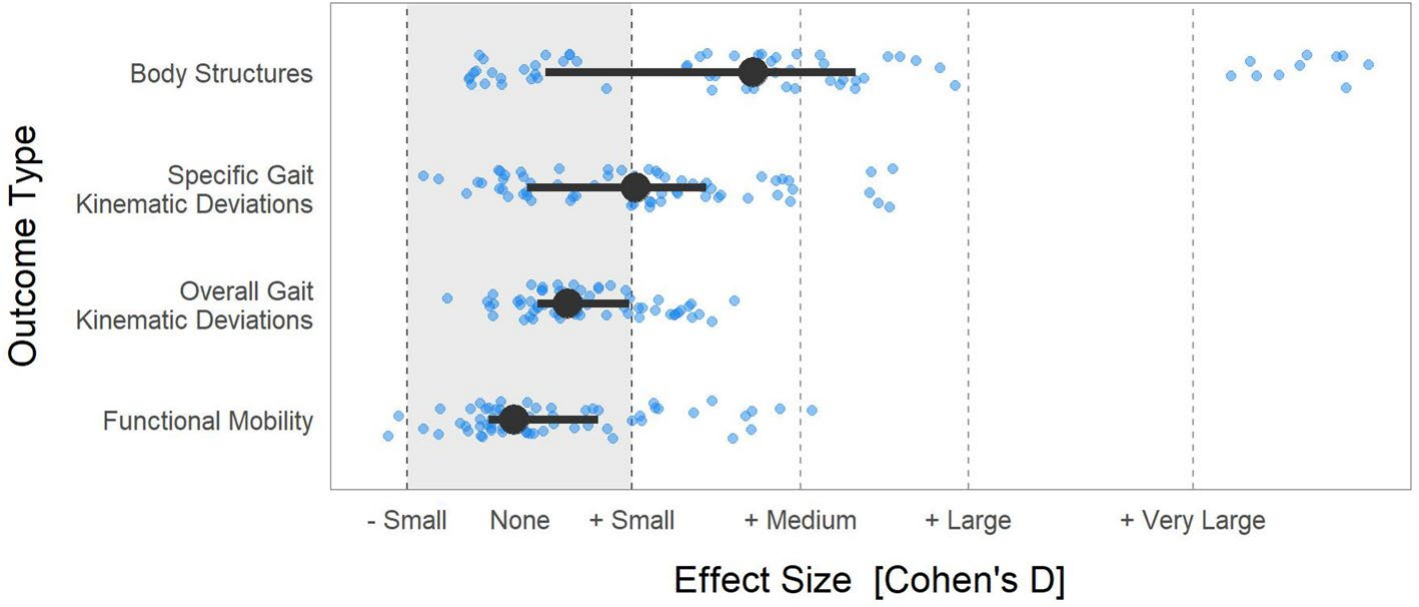
Median and interquartile range of short-term ATT across all treatments, models, and outcomes (large circles and horizontal lines) and individual model estimates across 5 models per outcome (small circles). We use conventional values for effect size thresholds (small ≥ 0.2, medium ≥ 0.5, large ≥ 0.8, very large ≥ 1.2). The very large effects are from Selective Dorsal Rhizotomy (mean spasticity) and Femoral Derotation Osteotomy (femoral anteversion).

**Figure 2:**
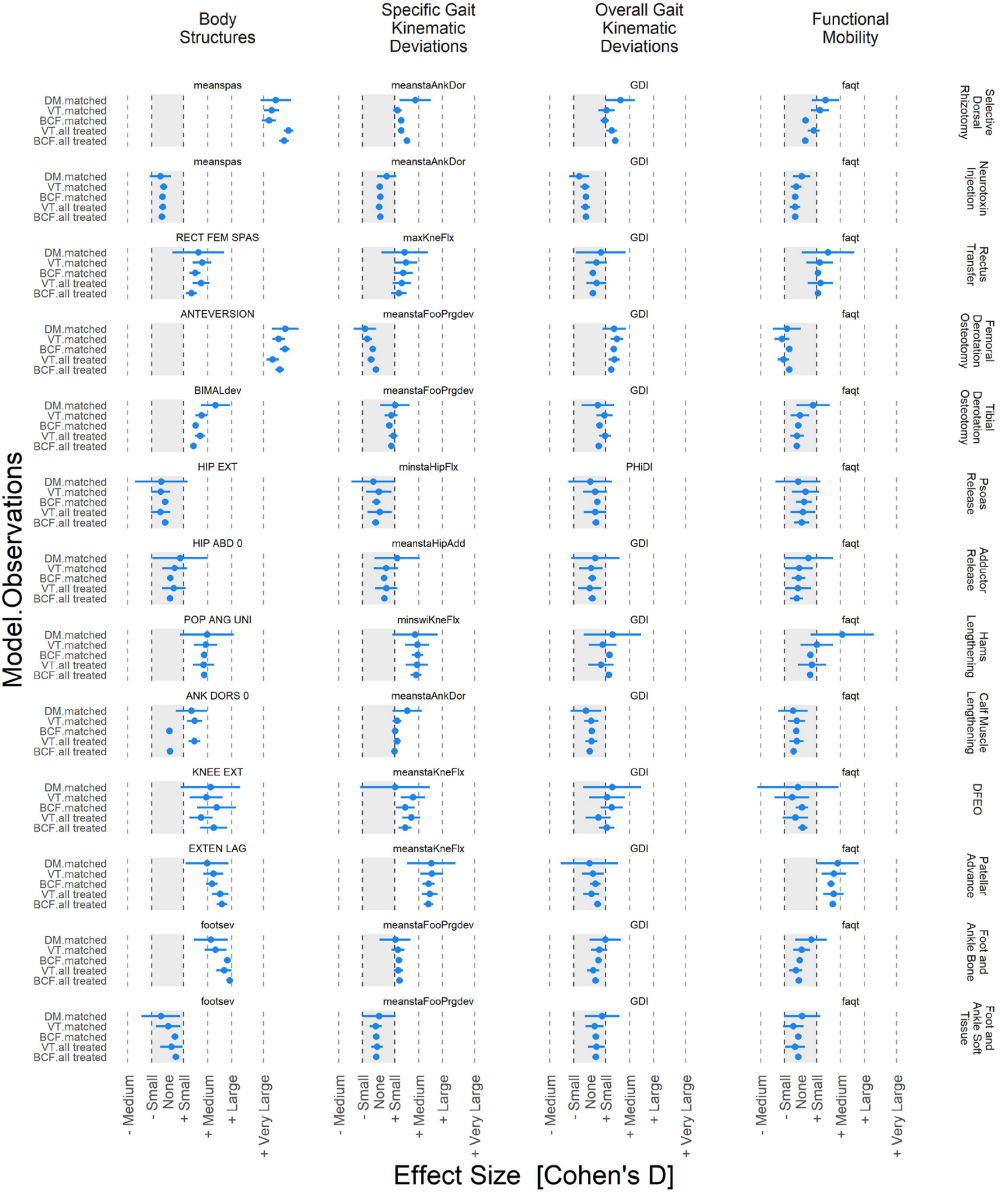
Effect sizes (mean and 95% CI) for 13 treatments, four outcome levels, three models, and two sets of observations. There is good consistency between models and between samples (matched subset and all treated) within a given model (VT or BCF).

In general, effects were largest at the body structures level (borderline medium effect), and decreased to borderline small or none as we moved along the causal chain through specific kinematic deviations, overall kinematic deviations, and functional mobility (Fig. 1). The ATT for each of the 13 treatments at each of the four levels show good consistency across models (Fig. 2).

There were a few model inconsistencies, such as change in mean stance ankle dorsiflexion after Selective Dorsal Rhizotomy or change in passive ankle dorsiflexion after calf muscle lengthening. However, in these cases the differences were modest, varying within the limits of a single effect category. There were no disagreements among any models, treatments, or outcomes in terms of the sign of the effect. Effect sizes for individual outcomes ranged from very large (e.g., spasticity reduction following selective dorsal rhizotomy) to no effect (many examples). There were no negative ATTs for any treatment at any level of outcome.

### Model performance

To assess the quality of the ATT estimates, we need to examine the performance of the DM, VT, and BCF models.

#### Direct matching

Matches were obtained for most limbs across all 13 treatments, (mean = 74%, range = 46–95%) of treated limbs (Table 2).

**Table 2:** Numbers of treated and matched observations.

Treatment	# treated	# matched	% matched
Selective dorsal rhizotomy	435	200	46
Neurotoxin injection	522	478	92
Rectus transfer	91	79	87
Femoral derotation osteotomy	440	302	69
Tibial derotation osteotomy	316	190	60
Psoas release	96	90	94
Adductor release	78	69	88
Hamstrings lengthening	86	76	88
Calf muscle lengthening	318	210	66
Distal femoral extension osteotomy	75	41	55
Patellar advancement	145	93	64
Foot and ankle: bone	336	189	56
Foot and ankle: soft tissue	147	137	93
Mean	237	166	74

The imposed criteria resulted in excellent matching of the physical exam, kinematic, and propensity score covariates, reducing standardized mean differences (SMD) between two- and six-fold (Table 3). Note that the SMD for factor variables was computed following Yang and Dalton^17^.

**Table 3:** Effect of matching on covariate balance (standardized mean difference).

Treatment	Kinematics		Propensity		Physical examination	
	Raw	Matched	Raw	Matched	Raw	Matched
Selective dorsal rhizotomy	0.22 (0.23)	0.08 (0.05)	0.60 (0.69)	0.11 (0.11)	0.30 (0.30)	0.09 (0.07)
Neurotoxin injection	0.08 (0.06)	0.05 (0.03)	0.23 (0.27)	0.05 (0.06)	0.11 (0.11)	0.06 (0.05)
Rectus transfer	0.25 (0.20)	0.10 (0.08)	1.09 (0.74)	0.13 (0.10)	0.35 (0.34)	0.12 (0.10)
Femoral derotation osteotomy	0.14 (0.13)	0.07 (0.05)	0.56 (0.50)	0.11 (0.08)	0.20 (0.22)	0.08 (0.06)
Tibial derotation osteotomy	0.14 (0.11)	0.07 (0.06)	0.69 (0.41)	0.10 (0.12)	0.19 (0.21)	0.08 (0.07)
Psoas release	0.23 (0.17)	0.14 (0.10)	0.99 (0.64)	0.14 (0.07)	0.33 (0.31)	0.15 (0.11)
Adductor release	0.34 (0.24)	0.10 (0.07)	1.05 (0.69)	0.12 (0.08)	0.41 (0.34)	0.12 (0.10)
Hamstrings lengthening	0.28 (0.21)	0.16 (0.13)	0.89 (0.60)	0.15 (0.09)	0.33 (0.30)	0.16 (0.13)
Calf muscle lengthening	0.12 (0.08)	0.08 (0.06)	0.55 (0.48)	0.14 (0.10)	0.18 (0.21)	0.09 (0.07)
DFEO	0.33 (0.29)	0.18 (0.13)	1.40 (1.33)	0.16 (0.19)	0.45 (0.52)	0.19 (0.16)
Patellar advancement	0.27 (0.24)	0.11 (0.08)	0.97 (0.78)	0.13 (0.10)	0.36 (0.35)	0.13 (0.10)
Foot and ankle: bone	0.16 (0.17)	0.12 (0.09)	0.69 (0.41)	0.13 (0.07)	0.21 (0.25)	0.11 (0.09)
Foot and ankle: soft tissue	0.14 (0.10)	0.08 (0.07)	0.63 (0.40)	0.17 (0.12)	0.20 (0.22)	0.11 (0.09)
Mean	0.21	0.10	0.79	0.13	0.28	0.11

All values mean (SD).

An important result from the DM model is the matching of prior and interval treatment. As noted above, the multi-level nature of surgery in CP means that most patients receive several simultaneous surgeries, many of which can be assumed to have treatment effects across all four levels of outcome (Table 4). The difference in rates (mean (SD)) of prior treatment was 3% (3%), and the difference in rates of interval treatment was 3% (3%).

**Table 4:** Effect of matching on balance of prior and interval treatment.

Interval treatment	Selective dorsal rhizotomy (%)	Neurotoxin injection (%)	Rectus transfer (%)	Femoral derotation osteotomy (%)	Tibial derotation osteotomy (%)	Psoas release (%)	Adductor release (%)	Hams length (%)	Calf muscle length (%)	DFEO (%)	Patella advance (%)	Foot ankle bone (%)	Foot ankle soft tiss (%)
**Difference in prior treatment rate (treated–control)**													
Selective dorsal rhizotomy	− 10	− 4	0	− 4	− 3	− 1	− 1	− 4	3	− 1	− 1	− 1	3
Neurotoxin injection	1	5	0	− 1	1	1	0	− 3	− 2	− 1	− 1	5	2
Rectus transfer	− 1	− 9	− 1	1	1	5	0	5	6	− 4	0	− 9	1
Femoral derotation osteotomy	− 7	− 3	1	− 2	− 2	− 1	− 1	− 1	− 1	2	− 1	− 2	− 1
Tibial derotation osteotomy	− 5	0	− 1	1	− 2	0	− 1	− 1	− 2	0	2	− 1	− 2
Psoas release	− 6	2	− 9	1	1	− 2	− 8	− 9	0	− 9	− 3	− 2	11
Adductor release	12	− 6	− 4	4	− 10	− 1	− 12	− 1	− 3	− 9	− 3	− 3	− 3
Hamstrings lengthening	− 7	0	4	8	13	− 4	1	1	8	0	0	− 3	− 1
Calf muscle lengthening	− 9	3	5	− 2	0	2	0	0	− 2	0	− 1	0	0
DFEO	0	− 2	2	17	0	2	0	− 5	0	20	0	− 2	15
Patellar advancement	8	− 4	− 2	3	− 3	9	− 2	8	9	− 4	2	8	− 5
Foot and ankle: bone	5	1	4	3	1	2	0	3	4	− 3	1	2	2
Foot and ankle: soft tissue	− 8	− 2	− 1	3	4	3	7	5	3	− 4	− 1	− 2	− 1
**Difference in interval treatment rate (treated–control)**													
Selective dorsal rhizotomy	100	− 2	− 2	− 2	− 2	− 2	− 1	− 1	− 2	− 2	− 2	− 2	− 2
Neurotoxin injection	− 1	100	1	7	1	0	0	0	2	0	1	1	0
Rectus transfer	− 3	0	100	3	− 3	4	3	3	5	4	4	4	4
Femoral derotation osteotomy	− 1	13	5	100	1	4	5	4	1	2	5	1	− 1
Tibial derotation osteotomy	− 1	− 5	2	1	100	4	6	3	1	− 2	4	3	1
Psoas release	− 2	− 7	19	2	3	100	2	− 1	2	2	2	2	13
Adductor release	− 3	12	9	4	4	4	100	3	0	4	4	4	− 1
Hamstrings lengthening	− 3	− 4	16	3	0	− 3	3	100	3	− 3	3	− 1	− 1
Calf muscle lengthening	− 1	0	− 1	1	1	1	− 1	1	100	− 1	− 1	1	1
DFEO	− 5	7	2	12	5	5	2	− 2	2	100	0	0	− 10
Patellar advancement	− 2	6	2	15	12	4	3	1	0	0	100	3	4
Foot and ankle: bone	− 1	− 6	1	1	2	1	− 2	1	1	1	1	100	2
Foot and ankle: soft tissue	− 1	− 2	11	− 1	2	7	4	− 2	1	− 1	1	1	100

#### BART, virtual twins, and Bayesian causal forests

The key mechanism by which the BART-based models (VT and BCF) provide unbiased ATT estimates is their ability to model a complex outcome response surface as a function of covariates^18^. Model accuracy is a surrogate for how well the VT and BCF models fit the response surface. For both the VT and BCF models we examined the predictive accuracy for the selected outcomes (Table 5). Detailed VT and BCF results are available in the electronic addenda.

**Table 5:** Performance of BART and BCF.

Outcome	Accuracy (root mean squared error)	
	BART	BCF
ANK_DORS_0	6.1	6.4
ANTEVERSION	7.5	9.4
BIMALdev	5.2	5.8
EXTEN_LAG	5.0	6.3
faqt (dimensionless)	11.1	11.4
footsev (dimensionless)	0.32	0.32
GDI (dimensionless)	6.5	7.0
HIP_ABD_0	5.6	6.0
HIP_EXT	7.9	8.2
KNEE_EXT	3.3	3.9
maxKneFlx	5.2	5.7
meanspas	0.17	0.23
meanstaAnkDor	3.5	4.5
meanstaFooPrgdev	5.8	6.7
meanstaHipAdd	3.9	3.9
meanstaKneFlx	5.6	6.4
minstaHipFlx	5.6	5.9
minswiKneFlx	6.1	6.7
PHiDI (dimensionless)	6.9	7.3
POP_ANG_UNI	8.5	9.3
RECT_FEM_SPAS (dimensionless)	0.27	0.36

All units degrees, except where otherwise noted.

Names left in raw form, see Appendix 1 for covariate name abbreviations.

## Discussion

The effects of 13 common treatments in children and young adults with CP were found to be generally small to medium at the body structures level (median [IQR] = 0.42 [0.05, 0.60]) and became smaller along the causal chain through specific kinematic deviations (0.21 [0.01, 0.33]), overall kinematic deviations (0.09 [0.03, 0.19]), and functional mobility (-0.01 [-0.06, 0.13]).

The fundamental problem with causal inference is that we can never simultaneously observe an individual under the actual and counterfactual treatment. As a result, we rely on theory and indirect evidence to provide support for the validity our estimates. We implemented modern, widely used, extensively validated approaches. Nevertheless, it is worth looking for indirect evidence of accuracy. There were two treatments that had large and very large ATTs: selective dorsal rhizotomy (reduction in mean spasticity, Cohen’s d $\sim$ 1.5) and femoral derotation osteotomy (reduction in anteversion, Cohen’s d $\sim$ 1.5). These ATTs are consistent with what has been found in observational studies as well as what can be reasonably surmised from the nature of the surgeries (sectioning nerve rootlets, large rotation of bone). In contrast, the models found no effect of neurotoxin injections, which is consistent with the very small or absent effects reported at 4–6 weeks post-injection, likely to be even smaller at a one year follow-up^19^.

Overall, positive effects (including borderline effects) at the body structures level were observed in 9 of 13 treatments. Since the body structures are being directly manipulated by the treatment, it is not surprising that we observe the largest ATTs at this level. Notable exceptions were neurotoxin injections, lengthening of the psoas or adductors, and soft tissue surgery at the foot and ankle. Foot and ankle soft tissue procedures (e.g., tendon transfers) are primarily prescribed for the correction of dynamic swing-phase foot deformity, so the lack of impact on static, weight-bearing, foot deformity measures is not surprising. Regarding psoas lengthening, previous analyses from our and other institutions have suggested this surgery is only marginally effective^20,21^. The adductor data are taken at face value, given that adductor lengthening is often prescribed to correct hip dislocation or subluxation, and there is little research into the effect of this procedure on gait related outcomes.

Overall, positive effects at the kinematic parameter level were observed in 8 of 13 treatments (including three borderline effects). Medium effects were observed for patellar advancement (mean stance knee flexion), followed by borderline medium effects from hamstrings lengthening (minimum swing-phase knee flexion). Small and borderline effects were observed for selective dorsal rhizotomy, rectus transfer, tibial derotation osteotomy, calf muscle lengthening, distal femoral extension osteotomy, and foot and ankle bony surgery. A notable result was the failure of a femoral derotation osteotomy to improve foot progression deviation. This was mostly due to a substantial number of limbs being “overcorrected” (i.e., exhibiting a post-treatment deformity in the opposite direction of the pre-treatment deformity). The risk for overcorrection of limbs has been previously noted, and seems to arise due to an over-reliance on static measures of anteversion in the absence of dynamic signs of internal rotation^22,23^.

Positive effects, including borderline effects at the overall kinematic deviations level, were observed in 5 of 13 treatments: selective dorsal rhizotomy, femoral derotation osteotomy, tibial derotation osteotomy, distal femoral extension osteotomy, and hamstrings lengthening. The first two are unsurprising, given their large to very large effect at the body structures level. The positive hamstrings results were unexpected. Historically, our institution has been conservative in the prescription of hamstrings lengthening surgery. Evidence of this can be seen in Arnold’s study of hamstring lengthening outcomes, where patients from our center comprised the control group of children meeting criteria for hamstrings lengthening but not receiving the treatment^24^. One possible explanation of the positive hamstrings result is that, for at least the last 24 years, our center has considered explicit muscle length and lengthening rate thresholds when evaluating candidacy for hamstrings lengthening^25^. While muscle length data cannot identify short hamstrings (rule in for surgery), they can identify hamstrings that attain adequate length during gait (rule out for surgery). Using this guidance prevents over-lengthening of non-contracted hamstrings. Importantly, in previous analyses of both psoas and calf muscle lengthening, the largest impact on outcomes arose from ruling out patients who were not good candidates for the surgery^20,26^. It seems reasonable to assume the same is true for the hamstrings.

Overall, positive effects at the functional mobility level (FAQt) were observed in 3 of 13 treatments, including two treatments with borderline effects. Patellar tendon advancement had a clear small effect, while rectus femoris transfer and hamstrings lengthening had borderline small effects. A femoral derotation osteotomy had a borderline negative effect—the closest any treatment came to having a negative ATT. While this was effect was not significant, it was surprising, and merits further investigation, given that femoral derotation osteotomy was the most common surgery in this dataset (22% of limbs).

The treatment effects reported here may not match the impressions of clinicians and scientists who work with this patient population. Part of this mismatch may arise from the fundamental difference between facts and opinions. Our opinions are influenced by many cognitive biases (e.g., representativeness heuristic) and logical fallacies (e.g., base rate neglect). Therefore, obtaining facts through measurement and analysis is critical. It is also important to reiterate that when outcomes are measured in an observational setting, rigorous causal inference techniques are needed since marginal distributions are often misleading. A causal analysis must account for, among other things, a proper control group, simultaneous treatments, and the effects of aging.

The direct matching algorithm worked exceptionally well, considering the complexity of matching physical examination, gait, and treatment covariates. The model was hand tuned to produce a balance between closeness of matching and number of matched observations. We prioritized matching of treatments, due to their importance in outcomes. We were able to achieve excellent results, matching both prior and interval treatments within 3%, on average. We also took care to maintain closely balanced baseline gait kinematic patterns, given the gait-centric nature of this study (mean SMD = 0.21). This was largely achieved with distance matching, though occasional moment matching parameters were required (e.g., to balance mean stance foot progression for calf muscle lengthening). We were able to match a majority (74%) of treated limbs. Concerns about bias due to omitted observations was addressed by including models that estimated the ATT for all treated limbs, not just the matched subset. Generally, we found no meaningful evidence of bias in the matching model. Direct matching produced wider confidence intervals than the other two methods, while also tending to produce slightly more positive effects sizes. The combination of these effects results in no meaningful difference in likelihood of positive treatment effects. It is worth reiterating that all propensity models and outcome prediction models used the same set of covariates. This was done in part for simplicity and uniformity of approach, and in part because it reflects the way in which treatment decisions are made in a “*holistic*” manner. Reviewing patient data consists of the iterative identification and assessment of problems based on different domains of the patient profile. It is not obvious which factors influence which treatments. Additionally, the use of a uniform set of covariates greatly simplifies future external efforts to refute or replicate the findings presented here.

With any retrospective study there is a possibility of selection bias. We defend against this with models that can generate ATT estimates on either all, or a subset of treated observations. We did not see meaningful differences between these observations. Another possible source of selection bias arises from only evaluating individuals seen for clinical gait analysis and returning for post-treatment evaluation. It is possible that these individuals have a different outcome from either patients not seen for gait analysis at all, or those not seen for follow-up evaluation. Reasonable arguments can be made that these omitted patients could fare better or worse than the sample we analyzed. In either case, it seems unlikely that such a bias would be large.

We used a comprehensive set of covariates, derived from extensive clinical experience and understanding of the underlying condition and mechanisms of treatment effect. We are limited, however, by the measures we routinely obtain for our patients, and this includes documentation of the patients’ individual goals. Some of our measures are noisy and possibly biased. The Ashworth score, for example, does not measure tone exclusively, and has questionable inter-rater reliability. Other ordinal measures, including strength, static motor control, GMFCS level, are susceptible to imprecision. The use of multidimensional matching and Bayesian tree-based estimators minimizes the biasing impact of these problems, though at the cost of wider variance in the resulting estimates. Also, we do not routinely and objectively measure certain potentially important factors, such as cognitive ability or socioeconomic status. There are logical and scientifically valid reasons to believe that these factors could play a meaningful role in treatment outcome. That said, we suspect that such factors are nearly randomly distributed among the treated and control observations we analyzed, and therefore would not introduce significant bias into our ATT estimates. This issue should be addressed in future work.

We chose a coarse definition of treatments, and therefore cannot examine the importance of varying techniques within a treatment category. In cases where we have studied technique differences within our center and across centers—such as selective dorsal rhizotomy at the conus medullaris versus the cauda equina or proximal versus distal femoral derotation osteotomy—we have not found meaningful differences on outcome^27,28^. Our definition of treatments focused on individual procedures, even though multi-level treatment is common. We suspect that there may be an additive effect in SEMLS, although noncomplementary combinations of treatments could exist as well. Applying the methods described here to SEMLS (two or more treatments on a given limb) and using no treatment as a control, we observed a median effect across all models and samples of 0.36 for GDI (between small and medium) and 0.02 for FAQt (no effect). Finally, the results presented here represent the short-term outcome at approximately one-year follow-up. Long-term and immediate impacts merit further investigation.

The replication crisis in science is real. Thus, generalizability to other centers needs to be tested. We intentionally chose commonly measured variables and broad treatment categories in service to this goal. We also used out-of-the-box R packages that are open-source, easy-to-use, fast, stable, and well documented. Finally, to aid other researchers, the electronic addenda to this manuscript include extensive and detailed descriptions of the modeling parameter choices and intermediate results. Detailed code and data can be provided upon reasonable request.

Communication to clinicians and patients remains a critical challenge for machine learning methods in medicine. It is important that the consumers of this information understand the strengths and weaknesses of the approaches so that they can make informed decisions based on the results. The DM approach is the easiest to understand and most intuitive of the three models. It was therefore included, even though it has the widest uncertainty bounds and is at greatest risk for bias due to omitted observations.

This study examined average effects for broad treatment categories. Finding factors that explain heterogeneous treatment effects (HTEs) is an obvious next step^29^. It is likely that effectiveness is influenced by patient factors like strength and motor control, treatment factors like whether a surgery is a revision, and clinician factors like surgeon experience. Beyond HTEs lies the last step in the most holy grail of personalized medicine—predicting individual treatment effects (ITEs). All three levels of analysis have strengths and weaknesses. While the ATT analysis is likely to be the most accurate and generalizable, it provides the least specific guidance for an individual patient. In contrast, ITEs could be valuable to the clinician and patient, but are likely to have extremely wide uncertainty bounds. Despite its limitations, the ATT level of forecasting is a significant improvement over the current norm in treatment of children and young adults with CP, which generally does not include any explicit guidance, and instead relies mostly on clinician experience, intuition, and local treatment culture.

## Methods

This study was reviewed and authorized by the University of Minnesota institutional review board review (STUDY00012420). All experiments were performed in accordance with relevant guidelines and regulations. Informed consent for use of medical records was obtained at the time of service from all participants or their legal guardian. An option to rescind this permission was offered to patients at every visit to our center.

All results from causal inference methods are conditional on modeling assumptions. In this study, we follow the principles of the Rubin causal inference framework^30,31^. We assume that by controlling for the appropriate set of causal pre-treatment covariates, either through matching (DM) or modeling (VT, BCF), observational data can be used to estimate the causal average treatment effect compared to an untreated control group. Choosing the proper set of covariates is, of course, the crucial decision in this approach, and will be described below.

### Participants and covariates

#### Participants

We queried our database for individuals diagnosed with CP, less than 25 years old, who had two standard clinical gait assessments at least nine months and less than 30 months apart. We considered limbs, rather than individuals, as observations. This was motivated by the asymmetry commonly observed in this patient population and the standard clinical process that generates treatment decisions based primarily on limb-level data. We used bootstrap confidence interval estimates to avoid making any assumptions about limb independence.

#### Covariates

A uniform set of covariates was chosen for all predictive models. We used the same covariates to derive propensity score models for each treatment. The propensity scores were used as inputs to the predictive models, and are particularly important for the BCF approach. The covariates used in the models describe diagnosis, anthropometry, time and distance parameters, neurological impairments, contracture, bony alignment, kinematic gait deviations, and both prior and interval treatment (Table 6). The baseline value of an outcome measure was always included as a covariate and is often a strong predictor of outcome.

**Table 6:** Covariates in causal models.

Category	Variables
Diagnosis	Topographic sub-type, diagnosis side, whether limb is affected or unaffected (unilateral sub-types)
Anthropometry	Age, sex
Time and distance parameters	Timing of foot-off, opposite foot-off, and opposite foot contact, dimensionless speed and step length (17)
Neurological Impairments	*Spasticity*: Modified Ashworth Scores for hip adductors, hip flexors, hamstrings, plantarflexors, and rectus femoris *Strength*: Manual Muscle Test Grades for hip abductors, hip flexors and extensors, knee flexors and extensors, and plantarflexors *Static selective motor control*: Clinical grade of absent, diminished, or typical control for hip abductors, hip flexors, hip extensors, knee flexors, knee extensors, and ankle plantarflexors
Contracture	*Ankle*: maximum passive ankle dorsiflexion (knee at 0° and 90° of flexion) *Knee*: maximum passive knee flexion and extension *Hip*: maximum passive hip flexion and extension
Bony alignment	*Tibial torsion*: bimalleolar axis angle *Femoral anteversion*: anteversion estimated by trochanteric prominence test, maximum hip internal and external rotation
Kinematic gait deviations	Gait deviation Index Discretized kinematic data for Levels × Planes × Measures, where * levels* = pelvis, hip, knee, ankle, and foot * planes* = sagittal, coronal, and transverse plane * measures* = angle value at initial contact and foot-off, angle value and timing of maximum and minimum during stance, swing, and overall, mean angle, angle range of motion during stance, swing, and gait cycle
Mobility-related function	Gross Motor Function Classification System (GMFCS) level
Prior and interval treatment	*Neurological*: selective dorsal rhizotomy, botulinum toxin type A or phenol injection, intrathecal baclofen pump implantation, other neurosurgery (e.g., shunt placement, neurectomy) *Bony*: femoral derotation osteotomy, tibial derotation osteotomy, foot and ankle bony surgery, distal femoral extension osteotomy *Soft tissue*: adductor release, foot and ankle soft tissue surgery, calf muscle lengthening, psoas release, hamstrings lengthening, patellar advance, rectus femoris transfer *Casting*: lower leg cast (short or long) *Propensity:* probability of receiving one of the 13 target treatments

The covariates were chosen pragmatically to span the patient factors that are measured, analyzed, and discussed when devising a treatment plan. We also limited measures to those that are likely to be obtained at most clinical gait centers in order to promote future efforts to replicate or refute the findings presented here. The variable names are largely self-explanatory, but a complete glossary is provided (Appendix 1).

### Gait and clinical examination measures

Three-dimensional gait kinematics were measured at baseline and follow-up. Kinematic deviations were computed as the mean of three to five barefoot over-ground walking trials collected at a self-selected speed. Our motion analysis laboratory is accredited by the Commission for Motion Laboratory Accreditation, used modern, three-dimensional gait analysis equipment and methodology, and employed highly experienced staff. Kinematics were computed using a modification of the Vicon Plug-in-Gait model (Vicon Motion Systems Ltd, UK) with hip centers and knee axes identified using functional methods, and malleoli identified using virtual markers^32,33^. Observations with knee varus-valgus range-of-motion > 15° were removed to enhance the quality of the transverse plane kinematic profile^34^.

Physical examinations were performed by licensed physical therapists. Spasticity was scored using the modified Ashworth scale^35^. Strength was estimated from a manual muscle test^36^. Static selective motor control at various levels was graded as absent, diminished, or typical. Range-of-motion was assessed passively using a hand-held goniometer.

### Function

Functional mobility was measured using the Functional Assessment Questionnaire Transform (FAQt)^37^. The FAQt is a difficulty-weighted average of the 23 mobility skills queried by the Functional Assessment Questionnaire^38^. The questionnaire is filled out by patients or parents, with no indication of who answered each question. The FAQt is strongly correlated with the Gross Motor Function Measure (r = 0.73).

### Missing data

There are valid reasons to believe that some missing data occur in meaningful clinical patterns. For example, it is common to find missing data among neurological covariates (strength, spasticity, selective motor control) in individuals with significant cognitive impairments, due to the patient’s inability to understand and follow directions. These same impairments are correlated with overall severity and may also impact treatment outcome due the child’s ability to participate fully in rehabilitation after surgery. Missing values in categorical data were assigned a value (“Miss”). This protects against data that are not missing (completely) at random. Missing values for the FAQt were imputed if $\ge$ 18/23 questions upon which the FAQt depends were present. The mice package in R was used for imputation of FAQt based on available FAQ skill values^39^.

### Propensity scores

Propensity scores used in the DM, VT, and BCF models were computed from separate BART models using the bartMachine package in R^40^. Propensity score modeling is not the focus of this paper, and many good methods exist for estimating propensity scores^41^. The propensity model performance on independent test set data is included for reference (Table 7).

**Table 7:** Performance of propensity score models.

Treatment	Acc	Sens	Spec	AUC
Selective dorsal rhizotomy	0.85	0.90	0.84	0.93
Neurotoxin injection	0.62	0.54	0.65	0.64
Rectus transfer	0.79	0.90	0.78	0.91
Femoral derotation osteotomy	0.77	0.76	0.78	0.85
Tibial derotation osteotomy	0.79	0.76	0.79	0.84
Psoas release	0.76	0.84	0.75	0.87
Adductor release	0.71	0.82	0.70	0.85
Hams lengthening	0.76	0.80	0.76	0.84
Calf muscle lengthening	0.77	0.70	0.78	0.82
Distal femoral extension osteotomy	0.88	0.96	0.88	0.98
Patellar advance	0.85	0.91	0.85	0.94
Foot and ankle bone	0.68	0.81	0.65	0.80
Foot and ankle soft tissue	0.68	0.68	0.68	0.73

*Acc* accuracy, *Sens* sensitivity, *Spec* specificity, *AUC* area under the receiver operation characteristic curve.

All results are for out-of-sample (independent) test data.

### Treatments and outcomes

At our center, the 13 treatments we will focus on in this study account for over 93% of the treatments performed on children and young adults seen for pre- and postoperative three-dimensional gait assessment (Table 8). These are consistent with the most common treatments performed in this population^42^. We have defined relatively broad treatment categories. For example, the treatment category “*calf muscle lengthening*” groups together a variety of different surgical techniques, such as Baker and Strayer. Our coarse-grained approach is intended to emphasize the “*big picture*” nature of this study. Differences in outcomes between sub-categories within a given treatment category (e.g., Baker vs. Strayer) are not considered here. Note that interval treatment includes all treatment between baseline and follow-up gait analysis. Interval treatment usually, but not always, occurs at a single event. Treatments are recorded in our database based on the patient’s medical record. Most, but not all, treatments occurred at our center.

**Table 8:** Treatments and Outcome measures.

Surgery	Body structures	Specific gait kinematic deviations
Selective dorsal rhizotomy	Mean spasticity^a^ (meanspas)	Mean stance ankle dorsiflexion
Neurotoxin injection	Mean spasticity	Mean stance ankle dorsiflexion
Femoral derotational osteotomy	Femoral anteversion	Foot progression deviation from typical (mean over stance)
Tibial derotational osteotomy	Bimalleolar axis angle deviation	Foot progression deviation from typical (mean over stance)
Foot and ankle bone surgery	Weight-bearing foot deformity severity^b^ (footsev)	Foot progression deviation from typical (mean over stance)
Distal femoral extension osteotomy	Knee extension	Mean stance knee flexion
Psoas release	Maximum hip extension	Minimum stance Hip Flexion
Hamstrings lengthening	Popliteal angle	Minimum swing knee flexion
Adductor lengthening	Hip abduction with knee extended	Mean stance hip abduction
Calf muscle lengthening	Ankle dorsiflexion with knee extended	Mean stance ankle dorsiflexion
Rectus femoris transfer	Rectus femoris spasticity^c^	Maximum swing knee flexion
Patellar advancement	Knee extensor lag	Mean stance knee flexion
Foot and ankle soft tissue surgery	Weight-bearing foot deformity severity	Foot progression deviation from typical (mean over swing)

^a^Mean Spasticity (meanspas) = Ashworth score averaged over adductors, hamstrings, rectus femoris, plantarflexors.

^b^Foot deformity severity (footsev) = numerical severity score (0-typical–3-severe) averaged over weight-bearing hindfoot and forefoot severity assessment.

^c^As measured by physical examination.

Outcomes were assessed at four levels for each treatment: body structures, specific gait kinematic deviations, overall gait kinematic deviations, and functional mobility. For all treatments except psoas release, overall kinematic deviations was measured by the gait deviation index (GDI) and functional mobility was measured by the FAQt^37^. For psoas release, overall kinematic deviations were measured by the pelvis-hip deviation index (PHiDI)^20^. Outcomes at the level of body structures and specific kinematic parameter were chosen for each treatment using clinical experience (Table 8).

### Models

All computations were performed in R^43^. We used the designmatch package for the DM estimate, the bartmachine package for the VT estimate, and the bcf package for the BCF estimate^12,40,44^.

#### Direct matching (DM)

For the DM approach, treatment effects are estimated from the difference in outcome between one-to-one matched treated and control observations. Matched controls are obtained by imposing the following constraints:

- **Distance**. Minimize the multivariate distance (Mahalanobis rank distance) between treated and control observation based on a set of relevant, treatment-specific physical examination and gait kinematic parameters. Penalize mismatches of propensity score (the probability of an observation undergoing a treatment, given a set of covariates) when they exceed a standardized mean difference of 0.2. This “*caliper*” on propensity score ensures that we match both the covariates and the propensity score.
- **Near-fine balance**. Match the groupwise distributions of treatments and, for certain treatments, key categorical physical examination measures that are not well balanced with distance matching alone.
- **Moment balance**. Match the means of relevant physical examination measures and gait kinematic parameters on a groupwise basis (treated vs. control).

For the DM estimate we used the bmatch function and the optimal subset approach, with the subset weight set to the median of the distance matrix and the glpk solver to find an approximate solution.

#### Virtual twins (VT)

For the VT approach we first built a predictive model of the outcome. Next, we generated a fabricated counterfactual version (virtual twin) of each observation. For example, if the observation was treated, the virtual twin was created by setting the treatment status to untreated while leaving all other covariates unchanged. An outcome prediction was then made on the virtual twin, and the treatment effect was computed as the difference between the actual and virtual twin outcomes. In our implementation of the VT estimate, we used Bayesian Additive Regression Trees (BART) as the predictive model using the bartMachine function with all default settings.

#### Bayesian Causal Forests (BCF)

In the BCF approach we used an underlying BART model, but in a manner substantially different from the VT approach. A BCF is a modification of the traditional BART that protects against targeted selection and the bias it can introduce^12^. Details can be found elsewhere, but the key innovation in the BCF model is to treat the predicted outcome as a sum of a treatment effect ($\tau$) plus the effect of other factors ($\mu$). In our context, the other outcome effect ($\mu$) arises from other treatments and patient natural history, such as the development of contracture, bony remodeling, neuromaturation, and growth. Both $\tau$ and $\mu$ are assumed to depend on a set of chosen covariates and the propensity score. For the BCF estimate we used the bcf function and all default settings except for ntree_moderate = 200 and base_moderate = 0.95. These were increased from their default values (50 and 0.25, respectively) since there is known to be substantial outcome heterogeneity across observations. We used 1000 burn-in Markov chain Monte Carlo iterations and 1000 iterations after burn-in.

#### Why use three models?

There is an extensive literature describing each of these models and their use. Of note for this study is the work of Hill, who demonstrated the principles by which BART-based models (e.g., VT and BCF) achieve accurate causal predictions^18^. This was followed up by the work of Dorie, who compared a large number of state-of-the-art causal inference methods on a large set of challenging datasets^45^. Dorie’s study showed that BART-based methods, including BCF, performed exceptionally well and provided more accurate and precise treatment predictions than other causal inference methods. The three methods described vary in approach—though they are not completely independent of one another. Each method also comes with certain assumptions and limitations. For example, the direct matching approach is the most easily understood, and most closely mirrors an RCT, but we can only estimate the treatment effect for treated observations that have a matching control. This may result in an effect estimate based on a small or potentially non-representative sample. In contrast, both the VT and BCF models can estimate a treatment effect on every treated observation. However, understanding the mechanism of estimation for the VT and BCF approaches requires significant statistical and algorithmic knowledge, and is harder to understand for clinicians and patients.

### Analysis

#### Sample considerations

The DM model produces a set of one-to-one matched treated and control observations (matched subset). A limitation of direct matching is that not every treated observation will have a matching control observation. The exclusion of treated limbs creates a risk of bias in the treatment effect estimate. For example, consider a hypothetical situation where more severely affected individuals benefit the most from a treatment but cannot be closely matched to untreated observations because all such severely affected individuals underwent treatment. We look for possible bias from this scenario by estimating a treatment effect for both the matched subset and all treated observations in the VT and BCF models. While the VT and BCF models can estimate effects for all observations, uncertainty in the regions of poor overlap tends to be large^18,45^.

#### Bootstrap bounds

For each model × treatment × outcome combination, the mean and 95% confidence interval for the average treatment effects were derived from 1000 bootstrap replicates sampled from the relevant sets of observations (matched subset or all treated). Our observations are limbs, so by using bootstrap estimates we avoid making assumptions about the strength of correlation between observations.

## Supplementary Information

Appendix 1.

Supplementary Information.
