# Supplementary material for: Short-term causal effects of common treatments in ambulatory children and young adults with cerebral palsy: three machine learning estimates
Source: Sci Rep. 2022 May 12;12:7818. doi: 10.1038/s41598-022-11875-5 (PMC9098860; doi:10.1038/s41598-022-11875-5)
Supplement: Supplementary file 1 — Appendix 1. [file 41598_2022_11875_MOESM1_ESM.pdf]

Appendix 1. Variable names

Domain	Variable Description	Variable Name
Diagnosis	topographic sub-type (i.e. hemiplegia, diplegia)	*dxmod*
	whether limb is affected (unilateral sub-types)	*affected*
Anthropometry	Age	*age*
	Sex	*sex*
Time and Distance	Timing of foot-off	*footoff*
	Timing of opposite foot-off	*oppfootoff*
	Timing of opposite foot contact	*oppfootcontact*
	Dimensionless speed	*NDspeed*
	Dimensionless step length	*NDsteplen*
Neurological Impairments	Modified Ashworth Scores for hip adductors	*ADDUCTOR_SPAS*
	Modified Ashworth Scores for hip flexors	*HIP_FLEX_SPAS*
	Modified Ashworth Scores for hamstrings	*HAMSTRING_SPAS*
	Modified Ashworth Scores for plantarflexors	*PLANTFLEX_SPAS*
	Modified Ashworth Scores for rectus femoris	*RECT_FEM_SPAS*
	Manual muscle strength grades for hip abductors	*HIP_ABD_STR*
	Manual muscle strength grades for hip flexors	*HIP_FLEX_STR*
	Manual muscle strength grades for hip extensors	*HIP_EXT_STR*
	Manual muscle strength grades for knee flexors	*KNE_FLEX_STR*
	Manual muscle strength grades for knee extensors	*KNE_EXT_STR*
	Manual muscle strength grades for ankle plantarflexors	*PLANTFLEX_STR*
	Static selective motor control for hip abductors	*HIP_ABD_SEL*
	Static selective motor control for hip flexors	*HIP_FLEX_SEL*
	Static selective motor control for hip extensors	*HIP_EXT_SEL*
	Static selective motor control for knee flexors	*KNE_FLEX_SEL*
	Static selective motor control for knee extensors	*KNE_EXT_SEL*
	Static selective motor control for ankle plantarflexors	*PLANTFLEX_SEL*
Contracture	Maximum ankle dorsiflexion (knee extended to 0°)	*ANK_DORS_0*
	Maximum ankle dorsiflexion (knee flexed to 90°)	*ANK_DORS_90*
	Maximum knee flexion	*KNEE_FLEX*
	Maximum knee extension	*KNEE_EXT*
	Unilateral popliteal angle	*POP_ANG_UNI*
	Maximum hip abduction (knee extended to 0°)	*HIP_ABD_0*
	Maximum hip abduction (knee flexed to 90°)	*HIP_ABD_90*
	Maximum hip flexion	*HIP_FLEX*
	Maximum hip extension	*HIP_EXT*
Bony Alignment	Bimalleolar axis angle	*BIMAL*
	Trochanteric prominence test (~femoral anteversion)	*ANTEVERSION*
	Maximum internal hip rotation	*HIP_INT_ROT*
	Maximum external hip rotation	*HIP_EXT_ROT*
Kinematic Gait Deviations	Gait Deviation Index	*GDI*
	Kinematic Parameters	*See below*
Overall Function	Gross motor function classification system	*gmfcs*

Prior and Interval	Selective dorsal rhizotomy	*Neural_Rhizotomy*
	Neurotoxin Injection (e.g. botulinum toxin, phenol)	*Neurotoxin_Injection*
	Intrathecal baclofen pump implantation	*Neural_Baclofen*
	Other neurosurgery (e.g. shunt placement, neurectomy)	*Neural_Other*
	Femoral derotation osteotomy	*Femoral_Derotation_Osteotomy*
	Tibial derotation osteotomy	*Tibial_Derotation_Osteotomy*
	Foot and ankle bony reconstruction	*Foot_and_Ankle_Bony*
	Distal femoral extension osteotomy	*DFEO*
	Adductor release	*Adductor_Release*
	Calf muscle lengthening	*Gastroc_Soleus_Lengthening*
	Psoas release	*Psoas_Release*
	Hamstrings lengthening	*Hams_Lengthening*
	Patellar Advancement	*Patellar_Advance*
	Rectus femoris transfer	*Rectus_Transfer*
	Foot and ankle soft tissue surgery	*Foot_and_Ankle_Soft_Tissue*
	Lower limb casting (long- or short-leg)	*Cast*
Propensity Scores	Propensity for treatment *TREAT*	*ps.TREAT*

Naming of kinematic parameters:

Kinematic sign conventions and abbreviations

Angle (Plane)	Abbreviation	Positive	Negative
Pelvic Tilt (Sagittal)	*PelTlt*	Anterior	Posterior
Pelvic Obliquity (Coronal)	*PelObl*	Up	Down
Pelvic Rotation (Transverse)	*PelRot*	Internal	External
Hip Flexion (Sagittal)	*HipFlex*	Flexion	Extension
Hip Adduction (Coronal)	*HipAdd*	Adduction	Abduction
Hip Rotation (Transverse)	*HipRot*	Internal	External
Knee Flexion (Sagittal)	*KneFlx*	Flexion	Extension
Knee Varus (Transverse)	*KneVar*	Varus	Valgus
Knee Rotation (Transverse)	*KneRot*	Internal	External
Ankle Dorsiflexion (Sagittal)	*AnkDor*	Dorsiflexion	Plantarflexion
Foot Progression (Transverse)	*FooPrg*	Internal	External

*Kinematic parameters are named by **level**, **plane**, and **measure**.*

- ***Level*** can be pelvis, hip, knee, ankle/foot
- ***Plane*** can be sagittal, coronal, or transverse
- ***Measure***

    - Angle (maximum, minimum, mean, initial contact value, foot-off value)
    - Timing of an angle (time of maximum, time of minimum),
    - Defined over stance-phase, swing-phase, or the entire gait cycle

Below are some examples

- ***Level*** = pelvis, ***Plane*** = coronal, ***Measure*** = minimum in stance

  minimum stance-phase pelvic obliquity (*minstaPelObl*).
- ***Level*** = knee, ***Plane*** = sagittal, ***Measure*** = timing of max in swing

  timing of maximum swing-phase knee flexion (*t_ maxswiKneFlx*)
- ***Level*** = hip, ***Plane*** = transverse, ***Measure*** = value at initial contact

  initial contact hip rotation (*icHipRot*)
- ***Level*** = foot, ***Plane*** = transverse, ***Measure*** = mean over gait cycle

  mean foot progression (*meanFooPrg*)

Note that there are no measures for ***Level*** = ankle and foot and ***Plane*** = coronal.
